# Effect of an Endurance and Strength Mixed Circuit Training on Regional Fat Thickness: The Quest for the “Spot Reduction”

**DOI:** 10.3390/ijerph18073845

**Published:** 2021-04-06

**Authors:** Antonio Paoli, Andrea Casolo, Matteo Saoncella, Carlo Bertaggia, Marco Fantin, Antonino Bianco, Giuseppe Marcolin, Tatiana Moro

**Affiliations:** 1 Department of Biomedical Sciences, University of Padua, 35131 Padua, Italy; antonio.paoli@unipd.it (A.P.); andrea.casolo@unipd.it (A.C.); matteo.saoncella@gmail.com (M.S.); carlo.bertaggia@gmail.com (C.B.); marco.fantin.3@studenti.unipd.it (M.F.); giuseppe.marcolin@unipd.it (G.M.); 2 Sport and Exercise Sciences Research Unit, University of Palermo, 90128 Palermo, Italy; antonino.bianco@unipa.it

**Keywords:** spot reduction, body composition, resistance training, adipose tissue

## Abstract

Accumulation of adipose tissue in specific body areas is related to many physiological and hormonal variables. Spot reduction (SR) is a training protocol aimed to stimulate lipolysis locally, even though this training protocol has not been extensively studied in recent years. Thus, the present study sought to investigate the effect of a circuit-training SR on subcutaneous adipose tissue in healthy adults. Methods: Fourteen volunteers were randomly assigned to spot reduction (SR) or to a traditional resistance training (RT) protocol. Body composition via bioimpedance analysis (BIA) and subcutaneous adipose tissue via skinfold and ultrasound were measured before and after eight weeks of training. Results: SR significantly reduced body mass (*p* < 0.05) and subcutaneous abdominal adipose tissue (*p* < 0.05). Conclusions: circuit-training SR may be an efficient strategy to reduce in a localized manner abdominal subcutaneous fat tissue depot.

## 1. Introduction

Regular physical activity can impact body composition, reducing fat mass and therefore positively improving health status. Accumulation of adipose tissue (AT) in specific areas of the body can be influenced by lifestyle behavior, such as working for most of the time in a sitting position or using only the upper body. Adipose tissue does not develop regularly but normally spread in distinctive anatomical depots [1]. Approximately 10–20% of total fat mass is contained in the visceral adipose tissue (VAT), located centrally and surrounds the internal organs [2]. The majority of total body fat is represented by the subcutaneous AT (SAT) positioned immediately below the skin: SAT normally accumulates in the gluteal, femoral and abdominal region and its distribution are regulated by different physiological and/or hormonal variables [3]. A small portion of AT consists in the ectopic AT and is localized around vital organs, such as the liver, heart and kidney.

The total amount of fat mass is considered a risk factor for several cardiometabolic diseases [4,5]; however, the location of lipids storage seems also to be critical for cardiometabolic consequences [6,7,8]. If central obesity is associated with metabolic dysfunction and hypertension [9,10], lower-body fat accumulation appears to have a protective effect and seems to be negatively correlated with cardiovascular disease and type 2 diabetes mellitus development [11,12]. The reduction of total body fat can be achieved through diet and/or exercise intervention [13,14]. While it has been widely demonstrated that an adequate amount of physical activity can have a favorable impact on the weight loss process [15], the existence of a “localized fat loss” is still on debate. As a matter of fact, for more than 60 years, the possibility of a localized removal of AT has raised interest in the scientific and social community. Even the “father” of the Mediterranean diet, Ancel Keys, admitted the possibility of a localized fat reduction, although not in a scientific journal, but rather on Vogue in 1956 [13]. Later, in the ‘50s, some researchers reported that certain sports, such as gymnast [16], basketball [17] or running [18], promoted greater loss of fat mass in those parts of the bodies that were vigorously exercised. Since then, different strategies have been developed to advance the localized loss of SAT with exercise, and all those protocols have been termed “spot reduction”. More recently, Stallknecht and coll. [19], hypothesized that exercise on specific muscles may induce “spot lipolysis” via an increased blood flow and release of fatty acids in the SAT nearby the contracting muscle regardless of exercise intensity. However, most of the studies found conflicting conclusions: some authors found a positive effect of spot reduction on localized lipolysis [20,21,22], while others were inconclusive [23,24,25,26]. The discrepancy between results can be found on the several exercise modalities employed, on the different body areas examined and, on the technique used for measuring SAT [27]. On the latter, most of the studies used skinfolds to evaluate changes after training [20,21,22,23,24,25]; recent comparative studies indicated that skinfold measurements do not permit accurate evaluation of SAT thickness because it is operator-dependent and influenced by anatomical site and skin thickness [28,29]. Regarding training modalities, it is well known that combining in the same training session endurance and strength exercises may exert a greater effect on total body fat loss [30] and, as recently demonstrated, and it might also have some positive effects on regional fat loss [31]. However, the effects of an alternation of strength and endurance training (mixed circuit training: MCT) has, until now, not been investigated. As a matter of fact, we demonstrated that a MCT induces greater total body fat loss and an improvement of metabolic variables compared to endurance training [32,33] but, except for our pilot trial in the 90′s [34] no one analyzed the effects of a MCT on regional fat loss.

In the light of the above, the purpose of the present study was to reconsider the spot reduction approach using a modified MCT protocol. MCT protocols usually alternate various total body strength exercises with short bouts of aerobic training. In the present study, we aimed to emphasize the positive effect of MCT by streamlining the order of proposed exercise to focus the major metabolic stress on the target body areas. To our best knowledge, this strategy has not been explored yet. We hypothesized that an MCT protocol concentrated on specific muscle would have exerted a great local lipolytic effect compare to a non-circuit mixed training. We tested our hypothesis on a group of healthy adults, using skinfolds and a modern ultrasound technique to measure SAT before and after eight weeks of training targeting abdominal and triceps.

## 2. Materials and Methods

This study is a randomized controlled parallel study. The study was approved by the ethical committee of the Department of Biomedical Sciences, University of Padova (HEC-DSB 05/17, 22 March 2017), according to the current Declaration of Helsinki. All participants read and signed a written informed consent form before enrollment.

Subjects were evaluated in a single visit before and after eight weeks of intervention. During the visit, height and body mass were measured, body composition was assessed via bioimpedance analysis (BIA) and skinfolds, and ultrasound was used to quantify the thickness of the adipose panicle. All measurements were taken by the same operator before and after the study. After the first screening visit, participants were randomized into two different groups: spot reduction (SR) or traditional resistance training (RT) and started the supervised training program.

### 2.1. Subjects

Eighteen volunteers (9 female and 9 male) aged between 20 and 46 years took part in the study. To be included in the protocol, subjects had to pass a medical interview and be aged 18–50 years old. Exclusion criteria were more than 1 year of training experience, chronic use of medication, metabolic disorders or any other clinical problems that could be aggravated by the study procedures or engagement in weight loss dietetic regimen. During the intervention, participants were allowed to continue their recreational physical activity, but were instructed not to perform any structured, high-impact training. Four subjects (two from each group) were excluded from the final analysis due to noncompliance with the training schedule. To be considered for the analysis, subjects had to complete all sessions and maintain a frequency of 3 training per week. Table 1 shows the anthropometric characteristics at baseline.

### 2.2. Measurements

Body mass index (BMI) was calculated in kg/m^2^, obtained from body mass and height measurement using a Wunder stadiometer (Holtain Ltd., Crymych, UK) with a precision of 0.1 kg and 1 cm, respectively.

Before proceeding with the measurements of the adipose panniculus by skinfolds and ultrasound, specific detection points were traced on the right portion of the body. For skinfolds, the 4 points described by the Durnin protocol [35] were used: bicipital, triceps, suprailiac and subscapularis. With regard to ultrasound scans, to standardize the procedure and detection points between subjects, we employed the protocol described by Muller et al. [36], and 8 regions were selected for the analysis: upper abdomen, lower abdomen, spinal erectors, distal triceps, brachioradialis, front thigh, medial calf, lateral thigh.

A mechanical caliper (GIMA, Gessate MI, Italy) was used to determined skinfolds to the nearest 1 mm. Each skinfold was measured 3 times, and the arithmetic means were used as the final value. Test–retest reliability for skinfold analysis was ICC = 0.96. Test–retest intra-observer reliability for fat adipose tissue thickness in our lab was ICC = 0.96, similar to previous findings [28,37].

Using the points previously marked, the skin fold was “pinched” between thumb and forefinger one centimeter above the measurement site, perpendicularly for the triceps and bicipital folds, and at a 45° angle to the longitudinal axis for the suprailiac and subscapular fold. All measurements were taken with the subject in an upright position and with the arms relaxed at the sides, while for the suprailiac point, the subject’s right arm was placed over the operator’s shoulder. Successively, body density was determined with Durnin–Womersley method [38], according to the methods Equation (1) was used to estimate body dansiy in male, whilst Equation (2) was used in female volounteers:

Male BD = 1.1765 − (0.744 × log_10_ Ʃ skinfolds); (1)

Female BD = 1.1567 − (0.0717 × log_10_ Ʃ skinfolds). (2)

Ultimately, body density from Equation (1) and (2) was used in Equation (3) to estimate body fat percentage using the Siri formula [39]:

FAT% = ((4.95/BD) − 4.5) × 100(3)

Ultrasound measurements were performed using Xario 100 ultrasound (TOSHIBA, Tustin, CA, US) with a surface probe set on MSK 1. The probe was equipped with a spirit level to maintain the same inclination in all readings. During acquisition, no pressure was ever exerted on the probe placed on the subject’s skin, except for the natural one resulting from the weight of the probe itself, by keeping the probe by the cable. For each detection point 3 snapshots were taken, following Muller’s procedures:

- Upper abdomen and Lower abdomen: subjects were positioned supine and asked to inhale and then to stop breathing at mid-exhalation to take the 3 photographs without movement of the abdominal wall;
- Brachioradialis: subjects were positioned supine, arms at the sides, right-hand with thumb up;
- Front thigh: subjects were positioned supine and asked to stay relaxed;
- Spinal erectors: subjects were prone, with the chin resting on the edge of the bed and arms extended at the sides;
- Distal triceps: subjects were prone, arms at the sides with right palm upwards;
- Lateral thigh: subject in lateral decubitus on the left side and legs at a 90° angle at the knee;
- Medial calf: subject in lateral decubitus on the right side, right leg with a 90° angle at the knee.

Body composition, total body water (TBW), extracellular (ECW) and intracellular (ICW) water, body cell mass (BCM) and phase angle (PA) were measured via bioelectrical impendence analysis (Akern, Body Pro, Pontassieve, Italy). Subjects were asked to empty their bladder and rest for ~3–5 min in a supine position, while four electrodes were placed on their hands and feet to start the analysis. Test–retest reliability for body composition analysis using bioelectrical impendence was ICC = 0.99.

### 2.3. Training Protocols

Subjects were required to perform the prescribed exercise protocol 3 times per week for a total of 8 weeks. The training was supervised by a certified trainer whose task was to check adherence to the study and the correct execution of training protocols.

Training protocols were comparable for the type of exercise, intensity, and volume but differed for the order of execution. The SR protocol was an alternation between endurance strength exercise (MCT) in which, specifically, abdominal, triceps and aerobic exercise were performed in a circuit (as described in Table 2) while the other muscular area (back, shoulders, arms, and lower limbs) were trained through RT exercises at the end of the circuit. RT group completed first all the aerobic exercises, and in the second part, the resistance exercises. Aerobic exercise intensity was settled at 65% of max HR using Cooper formula, while resistance load was assessed based on the previous training schedule and during a preliminary familiarization session.

### 2.4. Statistical Analysis

Results are presented as mean ± SD. The sample size was calculated based on preliminary data from our laboratory, assuming within-subject variability of 25% and a fixed power of 0.8 and an alpha risk of 0.05 for the main variables (skinfolds). Initially, the analysis revealed that 9 subjects per group were needed to achieve the above parameters. However, only 7 participants were included in the final evaluation; we thus perform a post hoc analysis and the achieved power with the real sample size was 0.75. An independent *t*-test was performed on baseline characteristics to ensure no difference between groups. After checking for normal distribution via the Shapiro–Wilk W test, a two-way ANOVA for repeated measures was performed to compare the effect of training modalities through a “time x training” analysis. The post hoc Bonferroni test was used to identify specific intragroup differences when suitable. For each group, Cohen’s d effect size was assessed by dividing the difference between mean values by the pooled SD. The *p*-value was set at 0.05. Data analysis was performed using GraphPad Prism software version 8.4.3 (GraphPad Software, San Diego, CA, USA).

## 3. Results

**Figure 1:**
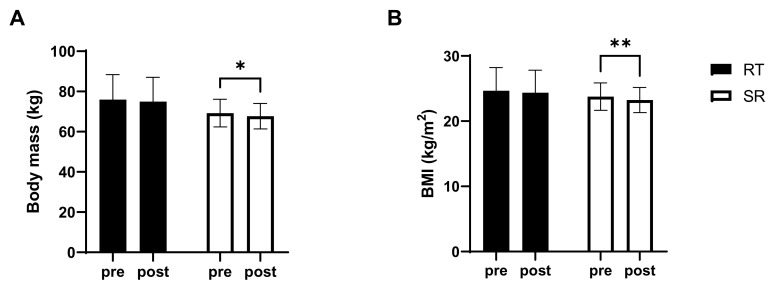
(**A**) body mass and (**B**) body mass index (BMI). RT resistance training group; SR, spot reduction group. * significantly different from pre-value (*p* < 0.05); ** significantly different from pre-value (*p* < 0.01).

Body mass significantly decreased (F(1,12) = 14.304; *p* = 0.003) in the SR group (from 69.24 ± 6.90 kg to 67.74 ± 6.34 kg; *p* = 0.01, d = −0.32), but not in the RT group (from 75.93 ± 12.47 kg to 74.96 ± 12.08 kg, *p* > 0.05, d = −0.11). As a consequence, also the BMI was significantly reduced (F(1,12) = 14.605; *p* = 0.002) only in the SR group (from 23.78 ± 2.11 kg/m^2^ to 23.27 ± 1.93 kg/m^2^, *p* = 0.01, d = −0.36) compare to RT group (from 24.67 ± 3.54 kg/m^2^ to 24.36 ± 3.44 kg/m^2^, *p* > 0.05, d = −0.13) as shown in Figure 1.

No differences in skinfolds were detected after RT protocol; while SR resulted particularly effective on suprailiac (−13.29%; *p* = 0.02, d = −0.56) and subscapularis (−7.59%, *p* = 0.04, d = −0.44) skinfold. In both sites, the two-way ANOVA analysis revealed a significant main Time effect (suprailiac: F(1,12) = 6.993; *p* = 0.01; subscapularis: F(1,12) = 5.822; *p* = 0.01). Furthermore, body fat percentage estimated with Siri equation presented a significant main effect of time (F(1,12) = 7.776; *p* = 0.02), with a significant decrease observed only in the SR group (*p* = 0.01, d = −0.21). Data on skinfolds results are shown in Table 3.

We observed a significant main time effect on ultrasound measurements of adipose panicle for upper abdomen (F(1,12) = 6.888; *p* = 0.02), spinal erectors (F(1,12) = 10.209; *p* = 0.01) and front thigh (F(1,12) = 5.855; *p* = 0.03) (Table 4). Post hoc test revealed a significant reduction only in the SR group for upper abdomen (−18.89%, *p* = 0.05, d = −0.66), spinal erector site (−19.45%, *p* = 0.04, d = −0.55).

Results from the body composition analysis via BIA are shown in Table 5. A significant time effect (F(1,12) = 5.776; *p* = 0.03) was observed only in the Intracellular body water, where RT resulted in a significant reduction (−7.89%, *p* = 0.04, d = −0.76). No other detectable differences were found.

## 4. Discussion

The study aimed to revisit the spot reduction training in the light of new methods to analyze SAT. We observed a significant general reduction of body mass and abdominal SAT, measured both with skinfold and ultrasound after 12 weeks of spot reduction training. Skinfold measurements also showed a reduction on the subscapularis site, while ultrasound revealed a decrease in the spinal erectors SAT.

Compared to ultrasounds, the skinfolds’ technique measures SAT within a compressed double layer of skin. Skin thickness may vary substantially among body area, for example, is lower in the upper arm compared to the abdomen [28,36], reducing the accuracy between measurements. A recent analysis revealed that ultrasound might overcome the problem linked to the compressibility and viscoelasticity of adipose tissue and thus represents a better tool to estimate SAT [36,40]. Despite the limitation mentioned, we decided to include skinfolds measurements to be consistent with most of the studies that have analyzed spot reduction protocols. It is also worth mentioning that the ultrasound technique relies on the operator performing the measurements as much as skinfolds. Despite trying to comply with all the standard procedures to reduce variability during measurements, we observed relatively large standard deviations of up to 5–6 times the observed difference. Overall, the pre-post analysis revealed a relative medium effect size (as suggested by the observed Cohen’s d > 0.5 for most measures) which may slightly weaken the validity of our findings.

Spot reduction is a training protocol aimed to reduce subcutaneous fat on a particular part of the body. The first protocols of spot reduction were created based on the assumption that the accumulation of fat in a specific body area is related to the activity of the adjacent muscles [20,22,41]. However, a better understanding of the mechanism of adipose accumulation/oxidation has revealed that this assumption might not be completely correct. Fatty acids taken from the diet are deposited in the adipose tissue based on hormonal and receptor action [42,43], like energy storage. During physical activity, muscle contraction demands energy; if the energy request is not solved with glycogen store, fats are mobilized from adipose tissue, released into the bloodstream, and carried to target cells to be oxidized. Lipolysis is mediated by hormonal fluxes (catecholamines, insulin and autocrine/paracrine factors), which reach adipose tissue passing through the circulatory network [42,43]. Circulating fatty acids can be provided from any body district, which does not necessarily must be involved with muscular effort; therefore, performing countless series only of a specific exercise may not be sufficient to promote lipolysis in that specific site. However, it was recently observed that lipolytic activity is associated with an increase of blood flow in the adipose tissue and, thus, to the oxygenation of the adipocyte, suggesting that “blood flow and lipolysis are generally higher in subcutaneous adipose tissue adjacent to contracting than adjacent to resting muscle irrespective of exercise intensity. Thus, specific exercises can induce “spot lipolysis” in adipose tissue” [19]. Based on these premises, the goal of spot reduction training should be to increase blood perfusion in the areas where it is most needed, which are where the adipose tissue is located; and sequentially promote fat oxidation. For this reason, the SR protocol we have employed in the present study was composed by a circuit training, in which the localized SAT mobilization was stimulated by target exercises (crunches for the abdomen and dumbbell overhead extension for the triceps), while fat oxidation was induced by the aerobic phases. Apart from our previous pilot study [34], this is the first attempt to use an MCT in an SR protocol.

We observed a significant reduction in the suprailiac skinfold and in the upper abdomen measure via ultrasound. These data are in accordance with others [20,21] and support the idea that spot reduction protocol can improve local lipolysis in the abdomen. We also observed a significant SAT reduction in the spinal erector site, which was adjacent to the subscapularis skinfold site, while we did not observe any direct effect on the triceps measurements. This was an unexpected result, as our hypothesis was that the specific triceps exercise included in the circuit training would have reduced the local SAT. We included tricipital exercise into our protocol because this is one of the areas in which subcutaneous adipose tissue can concentrate and also because triceps brachii can be exercised with several specific exercises. It is possible that, due to their inexperience, participants had involved more the shoulder and scapula-stabilizing muscles than the triceps brachialis during the execution of the dumbbell overhead extension exercise, reducing the effect on the specific site. It is also worth noting that, although not significantly, the front thigh SAT increased in the SR group. This result may be explained with a greater effort expressed from the participant during the first MCT part, which might have tired them out before facing the second part of the workout. It is, therefore, possible that lower limbs were not successfully trained. Although this is only speculation, and these results raise interesting future questions on the SR approach.

Overall, we found that SR reduced total body mass, while any significant difference was obtained after a traditional resistance training protocol. However, body compositional analysis via BIA was unable to detect any significant changes in total fat mass or lean body mass. We observed a significant decrease in the intracellular water after RT, which normally indicates alteration of the number and size of muscle cells; however, this did not reflect on lean body mass value. Using the Siri equation to estimate body fat percentage, we found a significant decrease in the SR group compared to the RT group. However, the Siri formula is dependent on the precision of skin-fold measurements, and generally, the error of this method is approximately 5% [44].

The two protocols contained the same exercises and were comparable for duration and volume. This implicates that to reduce body mass, training intensity is a more important variable than the type of exercise. Training intensity could be manipulated in several ways: by increasing loads or oxygen consumption level, but also reducing rest intervals or altering movement velocity. In the initial MCT part of the spot reduction protocol, subjects did not rest between exercises, which increased the overall training metabolic demand. This is in contrast with other studies comparing the general and localized type of exercise training [21,45], which found similar effects on body composition. However, in the mentioned studies, the two protocols did not match the intensity of training modalities; for example, Noland and coll. Compared a general aerobic training with a localized calisthenic-type exercise [21]; while Schade concentrated one protocol only in the hip and abdominal areas and expanded the generalized training adding exercises on the upper and lower body [45]. Finally, we hypothesized that the alternation of endurance and strength exercises, or put another way, the insertion of a strength training exercise for specific muscles inside an endurance training might enhance the reduction of the fat tissue adjacent to the exercising muscles.

A limitation of the present study was the reduced number of participants. Due to the variability between subjects, it would be important to increase the sample size in future studies to determine the effectiveness of spot reduction in a larger population.

## 5. Conclusions

Spot reduction training, conducted in a mixed circuit-training format (triceps and abdomen inside an endurance training), seems to be efficient in promoting adipose tissue reduction in the subcutaneous abdominal region, but was not efficient on the triceps site.

## Figures and Tables

**Table 1:** Baseline characteristics of spot reduction (SR) and resistance training (RT) groups.

	SR (*N* = 7)	RT (*N* = 7)	Difference between Group (*p*-Value)
Age (y)	23.29 ± 1.89	26.57 ± 9.14	0.37
Weight (kg)	69.24 ± 6.90	75.93 ± 12.47	0.24
Height (cm)	170.64 ± 5.73	175.57 ± 12.02	0.35
BMI (kg/m^2^)	23.78 ± 2.11	24.67 ± 3.54	0.58
Body fat (%)	24.69 ± 10.32	28.03 ± 7.44	0.50

All values are means ± SD.

**Table 2:** Training protocols.

SR			RT		
Exercise	Set × Reps/Time	Rest	Exercise	Set × Reps	Rest
Treadmill	5 min	-	Treadmill	5 min	-
Crunches	20 reps	-	Bike	5 min	-
Dumbbell overhead extension	15 reps	-	Step	5 min	-
Bike	5 min	-	Treadmill	5 min	-
Crunches	20 reps	-	Bike	5 min	-
Dumbbell overhead extension	15 reps	-	Dumbbell bench press	3 × 10	1 min
Step	5 min	-	Lat pulldown	3 × 10	1 min
Crunches	20 reps	-	Shoulder press	3 × 10	1 min
Dumbbell overhead extension	15 reps	-	Arm curl	3 × 12	45 s
Treadmill	5 min	-	Dumbbell overhead extension	4 × 15	45 s
Crunches	20 reps	-	Leg press	3 × 10	1 min 30 s
Dumbbell overhead extension	15 reps	-	Leg extension	3 × 12	1 min
Bike	5 min	-	Crunches	4 × 20	45 s
Dumbbell bench press	3 × 10	1 min	-	-	-
Lat pulldown	3 × 10	1 min	-	-	-
Shoulder press	3 × 10	1 min	-	-	-
Arm curl	3 × 12	45 s	-	-	-
Leg press	3 × 10	1 min 30 s	-	-	-
Leg extension	3 × 12	1 min	-	-	-

SR, Spot Reduction group; RT, Resostance Training group.

**Table 3:** Skinfold results of spot reduction (SR) and resistance training (RT) groups.

	SR (*N* = 7)		RT (*N* = 7)	
	Pre	Post	Pre	Post
Bicipital (mm)	11.66 ± 9.04	9.68 ± 5.09	8.50 ± 4.85	9.02 ± 6.14
Triceps (mm)	18.75 ± 9.31	18.65 ± 9.31	19.87 ± 5.36	19.81 ± 5.14
Suprailiac (mm) §	20.43 ± 6.01	17.99 ± 6.37 *	19.33 ± 6.39	18.85 ± 6.36
Subscapularis (mm) §	16.94 ± 5.74	15.36 ± 4.25 *	18.25 ± 6.47	17.17 ± 5.61
Body fat (%) §	28.83 ± 8.92	27.52 ± 8.97 *	27.74 ± 6.81	27.49 ± 6.91

All values are means ± SD. * significantly different from pre-value (*p* < 0.05); § significant time effect (*p* < 0.05).

**Table 4:** Ultrasound results of spot reduction (SR) and resistance training (RT) groups.

	SR (*N* = 7)		RT (*N* = 7)	
	Pre	Post	Pre	Post
Upper abdomen (mm) §	15.09 ± 6.08	12.35 ± 5.65 *	19.43 ± 10.34	18.13 ± 10.11
Lower abdomen (mm)	17.88 ± 7.44	17.30 ± 7.06	22.99 ± 9.23	22.33 ± 8.07
Spinal erectors (mm) §	9.96 ± 6.25	7.79 ± 4.83 *	10.77 ± 2.83	9.31 ± 3.28
Distal triceps (mm)	5.40 ± 3.95	6.21 ± 4.71	5.72 ± 2.13	5.19 ± 2.05
Brachioradialis (mm)	2.72 ± 1.37	3.24 ± 2.59	3.59 ± 2.12	3.66 ± 2.00
Front thigh (mm) §	8.46 ± 4.35	9.40 ± 5.17 *	11.56 ± 4.26	12.40 ± 3.29
Medial calf (mm)	4.87 ± 3.17	4.73 ± 2.90	5.90 ± 2.99	6.05 ± 2.49
Lateral thigh (mm)	18.44 ± 9.09	15.64 ± 7.08	24.10 ± 11.97	23.92 ± 11.08

All values are means ± SD. * significantly different from pre-value (*p* < 0.05). § significant time effect (*p* < 0.05).

**Table 5:** Bioimpedance analysis (BIA) results of spot reduction (SR) and resistance training (RT) groups.

	SR (*N* = 7)		RT (*N* = 7)	
	Pre	Post	Pre	Post
Total body water (L)	38.15 ± 6.09	36.74 ± 5.33	39.22 ± 6.22	38.36 ± 5.52
Extracellular water (L)	14.76 ± 1.97	13.93 ± 2.49	14.51 ± 2.76	14.41 ± 1.76
Intracellular water (L) §	23.38 ± 4.39	22.82 ± 3.79	24.71 ± 4.10	22.65 ± 3.63 *
Fat mass (kg)	17.13 ± 7.82	16.26 ± 6.57	21.42 ± 7.43	20.46 ± 8.12
Fat-free mass (kg)	52.15 ± 8.64	51.17 ± 7.27	54.58 ± 9.66	54.39 ± 9.45
Body cellular mass (kg)	28.85 ± 6.04	28.15 ± 5.53	30.91 ± 8.26	29.40 ± 6.85
Phase angle (°)	5.94 ± 0.56	5.91 ± 0.57	5.94 ± 1.17	5.66 ± 0.75

All values are means ± SD. * significantly different from pre-value (*p* < 0.05); § significant time effect (*p* < 0.05).

## Data Availability

Original data are available upon request to the corresponding author.

## References

[1] Walker G.E., Marzullo P., Ricotti R., Bona G., Prodam F. (2014). The pathophysiology of abdominal adipose tissue depots in health and disease. Horm. Mol. Biol. Clin. Investig..

[2] Despres J.P., Lemieux I. (2006). Abdominal obesity and metabolic syndrome. Nature.

[3] Smith S.R., Lovejoy J.C., Greenway F., Ryan D., de Jonge L., de la Bretonne J., Volafova J., Bray G.A. (2001). Contributions of total body fat, abdominal subcutaneous adipose tissue compartments, and visceral adipose tissue to the metabolic complications of obesity. Metabolism.

[4] Kivimaki M., Kuosma E., Ferrie J.E., Luukkonen R., Nyberg S.T., Alfredsson L., Batty G.D., Brunner E.J., Fransson E., Goldberg M. (2017). Overweight, obesity, and risk of cardiometabolic multimorbidity: Pooled analysis of individual-level data for 120 813 adults from 16 cohort studies from the USA and Europe. Lancet Public Health.

[5] Lu Y., Hajifathalian K., Ezzati M., Woodward M., Rimm E.B., Danaei G., Global Burden of Metabolic Risk Factors for Chronic Diseases Collaboration (2014). Metabolic mediators of the effects of body-mass index, overweight, and obesity on coronary heart disease and stroke: A pooled analysis of 97 prospective cohorts with 1.8 million participants. Lancet.

[6] Jensen M.D., Haymond M.W., Rizza R.A., Cryer P.E., Miles J.M. (1989). Influence of body fat distribution on free fatty acid metabolism in obesity. J. Clin. Investig..

[7] Alligier M., Meugnier E., Debard C., Lambert-Porcheron S., Chanseaume E., Sothier M., Loizon E., Hssain A.A., Brozek J., Scoazec J.Y. (2012). Subcutaneous adipose tissue remodeling during the initial phase of weight gain induced by overfeeding in humans. J. Clin. Endocrinol. Metab..

[8] Vogel M.A.A., Wang P., Bouwman F.G., Hoebers N., Blaak E.E., Renes J., Mariman E.C., Goossens G.H. (2019). A comparison between the abdominal and femoral adipose tissue proteome of overweight and obese women. Sci. Rep..

[9] Bosy-Westphal A., Geisler C., Onur S., Korth O., Selberg O., Schrezenmeir J., Muller M.J. (2006). Value of body fat mass vs anthropometric obesity indices in the assessment of metabolic risk factors. Int. J. Obes..

[10] Selvaraj S., Martinez E.E., Aguilar F.G., Kim K.Y., Peng J., Sha J., Irvin M.R., Lewis C.E., Hunt S.C., Arnett D.K. (2016). Association of Central Adiposity With Adverse Cardiac Mechanics: Findings From the Hypertension Genetic Epidemiology Network Study. Circ. Cardiovasc. Imaging.

[11] Seidell J.C., Perusse L., Despres J.P., Bouchard C. (2001). Waist and hip circumferences have independent and opposite effects on cardiovascular disease risk factors: The Quebec Family Study. Am. J. Clin. Nutr..

[12] Snijder M.B., Dekker J.M., Visser M., Bouter L.M., Stehouwer C.D., Kostense P.J., Yudkin J.S., Heine R.J., Nijpels G., Seidell J.C. (2003). Associations of hip and thigh circumferences independent of waist circumference with the incidence of type 2 diabetes: The Hoorn Study. Am. J. Clin. Nutr..

[13] Swift D.L., McGee J.E., Earnest C.P., Carlisle E., Nygard M., Johannsen N.M. (2018). The Effects of Exercise and Physical Activity on Weight Loss and Maintenance. Prog. Cardiovasc. Dis..

[14] Elagizi A., Kachur S., Carbone S., Lavie C.J., Blair S.N. (2020). A Review of Obesity, Physical Activity, and Cardiovascular Disease. Curr. Obes. Rep..

[15] Donnelly J.E., Blair S.N., Jakicic J.M., Manore M.M., Rankin J.W., Smith B.K., American College of Sports Medicine (2009). American College of Sports Medicine Position Stand. Appropriate physical activity intervention strategies for weight loss and prevention of weight regain for adults. Med. Sci. Sports Exerc..

[16] Cureton T. (1954). The Effect of Gymnastics upon Boys.

[17] Yuhasz M.S. (1962). The Effects of Sports Training on Body Fat in Man with Predictions of Optimal Body Weight.

[18] Kireilis R.W., Cureton T.K. (1947). The relationships of external fat to physical education activities and fitness tests. Res. Q. Am. Assoc. Healthphys. Educ. Recreat..

[19] Stallknecht B., Dela F., Helge J.W. (2007). Are blood flow and lipolysis in subcutaneous adipose tissue influenced by contractions in adjacent muscles in humans?. Am. J. Physiol. Endocrinol. Metab..

[20] Mohr D.R. (1965). Changes in Waistline and Abdominal Girth and Subcutaneous Fat Following Isometric Exercises. Res. Q..

[21] Noland M., Kearney J.T. (1978). Anthropometric and densitometric responses of women to specific and general exercise. Res. Q..

[22] Olson A.L., Edelstein E. (1968). Spot reduction of subcutaneous adipose tissue. Res. Q..

[23] Gwinup G., Chelvam R., Steinberg T. (1971). Thickness of subcutaneous fat and activity of underlying muscles. Ann. Intern. Med..

[24] Krotkiewski M., Aniansson A., Grimby G., Bjorntorp P., Sjostrom L. (1979). The effect of unilateral isokinetic strength training on local adipose and muscle tissue morphology, thickness, and enzymes. Eur. J. Appl. Physiol. Occup. Physiol..

[25] Roby F.B. (1962). Effect of exercise on regional subcutaneous fat accumulations. Res. Q. Am. Assoc. Healthphys. Educ. Recreat..

[26] Katch F.I., Clarkson P.M., Kroll W., McBride T., Wilcox A. (1984). Effects of sit up exercise training on adipose cell size and adiposity. Res. Q. Exerc. Sport.

[27] Kostek M.A., Pescatello L.S., Seip R.L., Angelopoulos T.J., Clarkson P.M., Gordon P.M., Moyna N.M., Visich P.S., Zoeller R.F., Thompson P.D. (2007). Subcutaneous fat alterations resulting from an upper-body resistance training program. Med. Sci. Sports Exerc..

[28] Muller W., Horn M., Furhapter-Rieger A., Kainz P., Kropfl J.M., Maughan R.J., Ahammer H. (2013). Body composition in sport: A comparison of a novel ultrasound imaging technique to measure subcutaneous fat tissue compared with skinfold measurement. Br. J. Sports Med..

[29] Bellisari A., Roche A.F., Siervogel R.M. (1993). Reliability of B-mode ultrasonic measurements of subcutaneous adipose tissue and intra-abdominal depth: Comparisons with skinfold thicknesses. Int. J. Obes. Relat. Metab. Disord..

[30] Villareal D.T., Aguirre L., Gurney A.B., Waters D.L., Sinacore D.R., Colombo E., Armamento-Villareal R., Qualls C. (2017). Aerobic or Resistance Exercise, or Both, in Dieting Obese Older Adults. N. Engl. J. Med..

[31] Scotto di Palumbo A., Guerra E., Orlandi C., Bazzucchi I., Sacchetti M. (2017). Effect of combined resistance and endurance exercise training on regional fat loss. J. Sports Med. Phys. Fit..

[32] Paoli A., Pacelli F., Bargossi A.M., Marcolin G., Guzzinati S., Neri M., Bianco A., Palma A. (2010). Effects of three distinct protocols of fitness training on body composition, strength and blood lactate. J. Sports Med. Phys. Fit..

[33] Paoli A., Pacelli Q.F., Moro T., Marcolin G., Neri M., Battaglia G., Sergi G., Bolzetta F., Bianco A. (2013). Effects of high-intensity circuit training, low-intensity circuit training and endurance training on blood pressure and lipoproteins in middle-aged overweight men. Lipids Health Dis..

[34] Dussini N., Martino R., Neri M., Paoli A., Velussi C. (1994). Multifactorial analysis of circuit-training induced regional fat reduction. Pflìugers Arch. Eur. J. Physiol..

[35] Durnin J., Rahaman M.M. (1967). The assessment of the amount of fat in the human body from measurements of skinfold thickness. Br. J. Nutr..

[36] Muller W., Horn M., Furhapter-Rieger A., Kainz P., Kropfl J.M., Ackland T.R., Lohman T.G., Maughan R.J., Meyer N.L., Sundgot-Borgen J. (2013). Body composition in sport: Interobserver reliability of a novel ultrasound measure of subcutaneous fat tissue. Br. J. Sports Med..

[37] Chirita-Emandi A., Dobrescu A., Papa M., Puiu M. (2015). Reliability of Measuring Subcutaneous Fat Tissue Thickness Using Ultrasound in Non-Athletic Young Adults. Maedica.

[38] Durnin J.V., Womersley J. (1974). Body fat assessed from total body density and its estimation from skinfold thickness: Measurements on 481 men and women aged from 16 to 72 years. Br. J. Nutr..

[39] Siri W.E. (1961). Body composition from fluid spaces and density: Analysis of methods. Tech. Meas. Body Compos..

[40] Ramirez M.E. (1992). Measurement of subcutaneous adipose tissue using ultrasound images. Am. J. Phys. Anthr..

[41] Checkley E. (1895). A Natural Material Method of Physical Education Training Making Muscle and Reducing Flesh without Dieting or Apparatus.

[42] Duncan R.E., Ahmadian M., Jaworski K., Sarkadi-Nagy E., Sul H.S. (2007). Regulation of lipolysis in adipocytes. Annu. Rev. Nutr..

[43] Jaworski K., Sarkadi-Nagy E., Duncan R.E., Ahmadian M., Sul H.S. (2007). Regulation of triglyceride metabolism. IV. Hormonal regulation of lipolysis in adipose tissue. Am. J. Physiol. Gastrointest. Liver Physiol..

[44] Lustig J., Strauss B. (2003). Nutritional Assessment. Anthropometry and Clinical Examination.

[45] Schade M., Helledrandt F., Waterland J.C., Carns M.L. (1962). Spot reducing in overweight college women: Its influence on fat distribution as determined by photography. Res. Q. Am. Assoc. Healthphys. Educ. Recreat..

